# Colonization With Extensively Drug-Resistant *Acinetobacter baumannii* and Prognosis in Critically Ill Patients: An Observational Cohort Study

**DOI:** 10.3389/fmed.2021.667776

**Published:** 2021-04-30

**Authors:** Yue Zheng, Nana Xu, Jiaojiao Pang, Hui Han, Hongna Yang, Weidong Qin, Hui Zhang, Wei Li, Hao Wang, Yuguo Chen

**Affiliations:** ^1^Shandong Provincial Clinical Research Center for Emergency and Critical Care Medicine, Institute of Emergency and Critical Care Medicine of Shandong University, Chest Pain Center, Qilu Hospital of Shandong University, Jinan, China; ^2^Department of Emergency Medicine, Qilu Hospital of Shandong University, Jinan, China; ^3^Key Laboratory of Emergency and Critical Care Medicine of Shandong Province, Key Laboratory of Cardiopulmonary-Cerebral Resuscitation Research of Shandong Province, Shandong Provincial Engineering Laboratory for Emergency and Critical Care Medicine, Qilu Hospital of Shandong University, Jinan, China; ^4^The Key Laboratory of Cardiovascular Remodeling and Function Research, The State and Shandong Province Joint Key Laboratory of Translational Cardiovascular Medicine, Chinese Ministry of Education, Chinese Ministry of Health and Chinese Academy of Medical Sciences, Qilu Hospital of Shandong University, Jinan, China; ^5^Cardiosurgery Care Unit, Department of Cardiosurgery, Qilu Hospital of Shandong University, Jinan, China; ^6^Department of Critical Care Medicine, Qilu Hospital of Shandong University, Jinan, China; ^7^Department of Clinical Laboratory, Qilu Hospital of Shandong University, Jinan, China; ^8^Department of Pharmacology, School of Basic Medical Sciences, Shandong University, Jinan, China

**Keywords:** *Acinetobacter baumannii*, extensively drug resistant, intensive care unit, colonization, infection

## Abstract

**Background:** *Acinetobacter baumannii* is one of the most frequently isolated opportunistic pathogens in intensive care units (ICUs). Extensively drug-resistant *A. baumannii* (XDR-AB) strains lack susceptibility to almost all antibiotics and pose a heavy burden on healthcare institutions. In this study, we evaluated the impact of XDR-AB colonization on both the short-term and long-term survival of critically ill patients.

**Methods:** We prospectively enrolled patients from two adult ICUs in Qilu Hospital of Shandong University from March 2018 through December 2018. Using nasopharyngeal and perirectal swabs, we evaluated the presence of XDR-AB colonization. Participants were followed up for 6 months. The primary endpoints were 28-day and 6-month mortality after ICU admission. The overall survival rate was estimated by the Kaplan-Meier method. We identified risk factors associated with 28-day and 6-month mortality using the logistic regression model and a time-dependent Cox regression model, respectively.

**Results:** Out of 431 patients, 77 were colonized with XDR-AB. Based on the Kaplan-Meier curve results, the overall survival before 28 days did not differ by colonization status; however, a significantly lower overall survival rate was obtained at 6 months in colonized patients. Univariate and multivariate analysis results confirmed that XDR-AB colonization was not associated with 28-day mortality, but was an independent risk factor of lower overall survival at 6 months (HR = 1.749, 95% CI = 1.174–2.608).

**Conclusions:** XDR-AB colonization has no effect on short-term overall survival, but is associated with lower long-term overall survival in critically ill patients.

## Introduction

*Acinetobacter baumannii* is one of the most important opportunistic pathogens in intensive care units (ICUs) and a major cause of nosocomial infections such as hospital-acquired pneumonia, peritonitis, and bacteremia (1–3). Characterized by its environmental resilience, communicability, and wide range of drug-resistance determinants, the infection and dissemination of *A. baumannii* in critical patients can pose a heavy burden on healthcare institutions (4–6).

Increased antimicrobial resistance of *A. baumannii* has become a worldwide challenge in the care of hospitalized patients. Extensively drug-resistant *A. baumannii* (XDR-AB) refers to strains that lack susceptibility to almost all antimicrobial agents except for one or two (e.g., polymyxin and tigecycline) (7). Previous studies have shown that 64.6% *A. baumannii* isolates are XDR or PDR in European countries (8), and the XDR prevalence is 73.1–80.6% in western Asian and South American countries (9, 10). In China, a national investigation program on microbial resistance reported that in 2004–2014, the prevalence of XDR-AB strains increased from 11.1 to 60.4% (11). Due to the limited availability of efficient treatments, XDR-AB infections contribute to extended hospitalizations and increased mortality rates (12, 13), which can reach 70% in critical patients with bacteremia (12).

Apart from symptomatic infections, *A. baumannii* colonizes hospitals and is commonly isolated from inpatient sites (14, 15). Due to the extraordinary environmental persistence and prevalence of extensive drug resistance, it is difficult to eradicate XDR-AB strains. Colonization may precede infections in severely ill patients and lead to poor prognoses. Carbapenemase-producing Enterobacteriaceae colonization has been proven to be associated with increased risk of mortality in ICU patients in a previous study (16). However, the effect of XDR-AB colonization in critically ill patients, especially its effect on long-term prognosis, is not clear. The aim of this study was to evaluate the impact of XDR-AB colonization on both the short-term and long-term mortality of critical patients.

## Methods

### Study Design

We conducted a prospective observational study in two mixed adult ICUs of Qilu Hospital of Shandong University from March 2018 through December 2018. Participants were followed up for 6 months after ICU admission. The study was approved by the Ethics Committee at Qilu Hospital. The need for informed consent was waived off.

### Participants

Patients admitted to ICUs were considered for the study. Patients were excluded if they (1) had a length of ICU stay <72 h; (2) were <18 years of age; (3) were lost to follow-up or had insufficient information; or (4) were assumed to have colonization prior to ICU admission. We recorded essential information of the patients (e.g., age, sex, diagnosis on admission, previous medical history, and comorbidities) upon ICU admission. We calculated the Charlson Comorbidity Index (CCI) to evaluate the mortality risk associated with comorbidities (17, 18). We used the Acute Physiology and Chronic Health Evaluation II (APACHE II) score (19) to assess the severity of illness. The APACHE II scores were calculated within 24 h of ICU admission. Administration of vasoactive drugs and mechanical ventilation was documented. XDR-AB infection during admission was assessed by an independent multidisciplinary panel of experts that included one infectious disease specialist and one intensivist.

### Microbiology

Nasopharyngeal and perirectal swabs were collected within 2 days after ICU admission for microbial culture and twice a week thereafter until a positive XDR-AB culture was obtained. If the patient stayed for more than 3 weeks, culture frequency was reduced to once a week. All samples were analyzed at the hospital's laboratories according to standard operating procedures. We performed strain identification and drug sensitivity testing with the VITEK2 COMPACT automatic microbial analysis system (bioMérieux, Marcy-l'Étoile, France). The susceptibility test breakpoints were based on the standards established by the American Clinical and Laboratory Standards Institute (CLSI) (20). *A. baumannii* (genospecies 2) was identified based on the 16S−23S ribosomal RNA gene intergenic spacer region (21).

### Definitions

We defined XDR-AB according to the guidelines of the US Centers for Disease Control and Prevention and World Health Organization (WHO) (7). Colonization with XDR-AB was defined as a positive XDR-AB culture during the ICU stay. If the first culture of XDR-AB is positive, it is assumed that the colonization occurred before ICU admission. Colonization is assumed if a negative culture is obtained before a positive one (16). The variables associated with mortality included age, sex, seasons, comorbidities and Charlson Index, admission source, primary reasons for ICU admission, intensity of care (renal replacement therapy, invasive ventilation, and vasopressor treatment), and APACHE II score.

### Statistical Analyses

We used SPSS 16.0 (SPSS Inc, IL, USA) and R software v3.0 for data analysis. The primary endpoint was overall survival at 28 days and 6 months after ICU admission. Quantitative data with normal distribution were expressed as mean ± standard deviations and compared using the Student *t*-test. Non-normally distributed data were analyzed using Wilcoxon's rank–sum test. Categorical variables were expressed as frequency (percentage) and compared using the Chi-square or Fisher's exact test (two-tailed). Overall survival was estimated by the Kaplan-Meier method, and the difference between Kaplan-Meier survival curves was evaluated using the log-rank test. A logistic regression model was used to explore the risk factors associated with XDR-AB colonization and 28-day mortality. Variables identified in univariate analysis with *P*-values <0.1 were included in the multivariate model. The Cox non-proportional hazards survival regression model was used for analyzing variables associated with mortality at 6 months. Potential time-dependent variables were tested using the proportional hazards assumption (Supplementary Table 1). XDR-AB colonization was treated as a time-dependent variable in the Cox regression model. Variables identified in univariate analysis with *P*-values <0.1 were included in the multivariate Cox regression models. *P* <0.05 was considered statistically significant according to multivariate analysis. We performed subgroup analysis to assess the consistency of the colonization effect on survival according to the baseline variables. The Cox proportional hazards model with Efron's method of handling ties was used to assess the magnitude of differences in XDR-AB colonization between patients with different baseline characteristics.

## Results

### Characteristics of Participants

Out of the initial population, 237 patients were excluded, including 191 patients with expected length of stay less than 3 days, 34 patients with no culture or culture results, 6 patients lost to follow-up or with insufficient data, and 6 patients in whom the colonization was assumed to have occurred before ICU admission. A total of 431 patients were finally included in the analyses. Out of these patients, 77 (17.87%) had XDR-AB colonization. Table 1 presents the baseline characteristics and outcomes of the participants. The participants had a median age of 60 years and were mostly male (68%). The overall 28-day and 6-month mortality rates were 15.5 and 48.0%, respectively.

**Table 1:** Descriptive results of potential risk factors and outcomes.

**Variables**	**Overall (*n* = 431)**	**XDR-AB Colonized (*n* = 77)**	**Not XDR-AB Colonized (*n* = 354)**	***P*-value**
**Age (years), median (IQR)**	60.15 (49, 72)	60.36 (50.5, 71.5)	60.11 (49, 73)	0.919
**Male**, ***n*** **(%)**	292 (67.7)	56 (72.7)	236 (66.7)	0.302
**Season**, ***n*** **(%)**				
Spring	147 (34.1)	24 (31.2)	123 (34.7)	0.907
Summer	103 (23.9)	18 (23.4)	85 (24.0)	0.811
Autumn	75 (17.4)	15 (19.5)	60 (16.9)	0.497
Winter	106 (24.6)	20 (26.0)	86 (24.3)	0.599
**Charlson Index, mean** **±** **SD**	4.22 ± 2.146	4.52 ± 2.180	4.16 ± 2.136	0.181
**Comorbidities**, ***n*** **(%)**				
Cardiovascular diseases	166 (38.5)	41 (53.2)	125 (35.3)	0.003
Chronic renal insufficiency	61 (14.2)	10 (13.0)	51 (14.4)	0.746
COPD	95 (22.0)	24 (31.2)	71 (20.1)	0.033
Type II diabetes mellitus	101 (23.4)	28 (36.4)	73 (20.6)	0.003
Solid tumor	106 (24.6)	10 (13.0)	96 (27.1)	0.009
Hematologic malignancy	8 (1.9)	2 (2.6)	6 (1.7)	0.595
Current or former smoker	160 (37.1)	35 (45.5)	125 (35.3)	0.095
**Admission source**, ***n*** **(%)**				
Emergency department	110 (25.5)	17 (22.1)	93 (26.3)	0.178
Operation theater	106 (24.6)	18 (23.4)	88 (24.9)	0.936
Normal wards	126 (29.2)	19 (24.7)	107 (30.2)	0.761
Other hospital	89 (20.6)	23 (29.9)	66 (18.6)	0.072
**Primary reason for ICU admission**, ***n*** **(%)**				
Neurological	53 (12.3)	5 (6.5)	48 (13.6)	0.087
Suspected sepsis	101 (23.4)	28 (36.4)	73 (20.6)	0.003
Respiratory	129 (29.9)	25 (32.5)	104 (29.4)	0.592
Cardiovascular (excluding stroke)	65 (15.1)	11 (14.3)	54 (15.3)	0.830
Multiple trauma	25 (5.8)	3 (3.9)	22 (6.2)	0.430
Burns	7 (1.6)	3 (3.9)	4 (1.1)	0.082
Hepatobiliary and pancreatic	21 (4.9)	0 (0.0)	21 (5.9)	0.028
Gastrointestinal	19 (4.4)	1 (1.3)	18 (5.1)	0.142
Genitourinary	11 (2.6)	1 (1.3)	10 (2.8)	0.442
**APACHE II score, mean** **±** **SD**	15.278 ± 8.837	19.273 ± 8.871	15.409 ± 8.600	<0.001
**Intensity of care**, ***n*** **(%)**				
Renal replacement therapy	71 (16.5)	20 (26.0)	51 (14.4)	0.013
Invasive ventilation for more than 5 days	209 (48.5)	64 (83.1)	145 (41.0)	<0.001
Vasopressor treatment for more than 3 days	100 (23.2)	28 (36.4)	72 (20.3)	0.003
**XDR-AB infection**, ***n*** **(%)**	30 (7.0)	17 (22.1)	13 (3.7)	<0.001
**Clinical outcomes**				
Length of stay in ICU, median (IQR)	18.85 (3, 83)	28.58 (3,76)	16.74 (3,83)	<0.001
28-day mortality, *n* (%)	67 (15.5)	12 (15.6)	55 (15.5)	0.992
Six-month mortality, *n* (%)	207 (48.0)	53 (68.8)	154 (43.5)	<0.001

*Data are number (%), median (IQR), or mean ± SD. XDR-AB, extensively drug-resistant Acinetobacter baumannii; COPD, chronic obstructive pulmonary disease; ICU, intensive care unit; APACHE, Acute Physiology and Chronic Health Evaluation; IQR, interquartile range; SD, standard deviation*.

Patients who tested positive for XDR-AB were more likely to have cardiovascular diseases, type II diabetes mellitus, solid tumors, suspected sepsis, supportive treatment (e.g., renal replacement therapy, longer invasive ventilation, and vasopressor treatment), and XDR-AB infections. Compared to non-colonized patients, XDR-AB colonized patients had higher APACHE II scores and 6-month mortality rates (68.8 vs. 43.5%; *P* < 0.001) and longer ICU days. However, 28-day mortality did not differ between colonized and non-colonized patients (15.6 vs. 15.5%, respectively; *P* = 0.992; Table 1). Multivariate logistic analysis indicated that suspected sepsis, extended invasive ventilation, and length of stay in the ICU were risk factors for XDR-AB colonization (Supplementary Table 2).

### Kaplan-Meier Analysis of Overall Survival With Colonization Status

**Figure 1:**
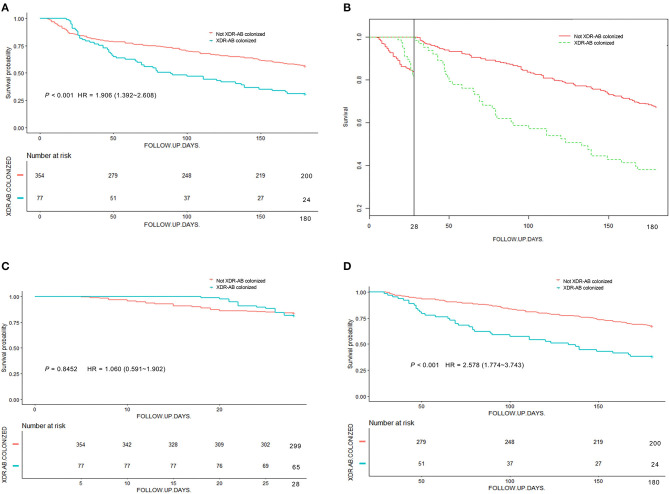
Kaplan-Meier survival analysis stratified by XDR-AB colonization and no colonization. Survival of patients was followed for 6 months. **(A)** Kaplan-Meier analysis of survival in colonized and non-colonized patients. **(B)** Survival curves before and after 28 days. **(C,D)** Landmark analysis discriminating between events occurring before and after 28 days of follow-up.

Figure 1 shows that the 6-month overall survival rate was higher for non-colonized patients than for XDR-AB-colonized patients (*P* < 0.001). In the analysis of events occurring before and after the 28-day follow-up, we observed that the overall survival rate before 28 days did not differ based on the colonization status (*P* = 0.845). However, the 6-month overall survival rate was significantly lower in colonized patients after 28 days (*P* < 0.001).

### Factors Predictive of 28-day Mortality

The risk factors for 28-day mortality identified through univariate analysis include CCI, APACHE II score, ICU admission with cardiovascular diseases, support with renal replacement therapy, and extended invasive ventilation (*P* < 0.100 for all, Table 2). The multivariate logistic regression model showed that only the APACHE II score was an independent risk factor for 28-day mortality (OR = 1.114, 95% CI = 1.076–1.154; *P* < 0.001; Table 3).

**Table 2:** Univariate analysis of factors associated with 28-day mortality.

**Variables**	**Deaths at day 28 (*n* = 67)**	**Survivals at day 28 (*n* = 364)**	***P*-value**	**OR (95%*CI*)**
**Age, median (IQR)**	61.42 (53,74)	59.92 (49,72)	0.509	1.005 (0.990–1.021)
**Male**, ***n*** **(%)**	42 (62.7)	250 (68.7)	0.336	1.305 (0.759–2.245)
**Season**, ***n*** **(%)**				
Spring	25 (37.3)	122 (33.5)	0.547	1.181 (0.687–2.028)
Summer	11 (16.4)	92 (25.3)	0.122	0.581 (0.292–1.156)
Autumn	8 (11.9)	67 (18.4)	0.203	0.601 (0.274–1.317)
Winter	23 (34.3)	83 (22.8)	0.046	1.770 (1.010–3.100)
**Charlson Index, mean** **±** **SD**	4.640 ± 1.747	4.150 ± 2.205	0.084	1.104 (0.987–1.235)
**Comorbidities**, ***n*** **(%)**				
Cardiovascular diseases	28 (41.8)	138 (37.9)	0.549	1.176 (0.692–1.997)
Chronic renal insufficiency	13 (19.4)	48 (13.2)	0.183	1.585 (0.805–3.120)
COPD	19 (28.4)	76 (20.9)	0.177	1.500 (0.833–2.701)
Type II diabetes mellitus	17 (25.4)	84 (23.1)	0.684	1.133 (0.621–2.069)
Solid tumor	17 (25.4)	89 (24.5)	0.872	1.051 (0.577–1.914)
Hematologic malignancy	1 (1.5)	7 (1.9)	0.811	0.773 (0.094–6.384)
Current or former smoker	20 (29.9)	140 (38.5)	0.182	0.681 (0.387–1.197)
**Admission source**, ***n*** **(%)**				
Emergency department	21 (31.3)	89 (24.5)	0.236	1.411 (0.799–2.491)
Operation theater	14 (20.9)	92 (25.3)	0.445	0.781 (0.414–1.473)
Normal wards	21 (31.3)	105 (28.8)	0.680	1.126 (0.641–1.979)
Other hospital	11 (16.4)	78 (21.4)	0.353	0.720 (0.360–1.441)
**Primary reason for ICU admission**, ***n*** **(%)**				
Neurological	7 (10.4)	46 (12.6)	0.617	0.807 (0.348–1.871)
Suspected sepsis	14 (20.9)	87 (23.9)	0.594	0.841 (0.445–1.589)
Respiratory	20 (29.9)	109 (29.9)	0.988	0.996 (0.563–1.759)
Cardiovascular(excluding stroke)	16 (23.9)	49 (13.5)	0.031	2.017 (1.066–3.814)
Multiple trauma	2 (3.0)	23 (6.3)	0.295	0.456 (0.105–1.982)
Burns	1 (1.5)	6 (1.6)	0.926	0.904 (0.107–7.632)
Hepatobiliary and pancreatic	2 (3.0)	19 (5.2)	0.441	0.559 (0.127–2.457)
Gastrointestinal	2 (3.0)	17 (4.7)	0.540	0.628 (0.142–2.784)
Genitourinary	3 (4.5)	8 (2.2)	0.287	2.086 (0.539–8.073)
**XDR-AB colonization**, ***n*** **(%)**	12 (17.9)	65 (17.9)	0.992	1.004 (0.509–1.980)
**XDR-AB infection**, ***n*** **(%)**	7 (10.4)	23 (6.3)	0.227	1.730 (0.711–4.210)
**Acuity score on admission**				
APACHE II score, mean ± SD	22.475 ± 6.647	13.953 ± 8.554	<0.001	1.114 (1.078–1.150)
**Intensity of care**, ***n*** **(%)**				
Invasive ventilation for more than 5 days	39 (58.2)	170 (46.7)	0.085	1.589 (0.938–2.693)
Renal replacement therapy	16 (23.9)	55 (15.1)	0.078	1.763 (0.938–3.311)
Vasopressor treatment for more than 3 days	67 (100)	33 (9.1)	0.992	3.279 (0.978–7.923)

*Data are number (%), median (IQR) or mean ± SD. OR, odds ratio; COPD, chronic obstructive pulmonary disease; ICU, intensive care unit; XDR-AB, extensively drug-resistant Acinetobacter baumannii; APACHE, Acute Physiology and Chronic Health Evaluation; IQR, interquartile range; SD, standard deviation*.

**Table 3:** Multivariate Logistic regression of Factors Associated with 28-day mortality.

**Variables**	**OR (95% *CI*)**	***P*-value**
XDR-AB colonization	0.517 (0.237–1.128)	0.098
APACHE II score	1.114 (1.076–1.154)	<0.001
Charlson Index	1.086 (0.951–1.239)	0.222
Cardiovascular (excluding stroke)	1.097 (0.519–2.318)	0.809
Invasive ventilation for more than 5 days	1.295 (0.718–2.337)	0.390
Renal replacement therapy	1.032 (0.500–2.132)	0.932

*OR, odds ratio; CI, confidence interval; XDR-AB, extensively drug-resistant Acinetobacter baumannii; APACHE, Acute Physiology and Chronic Health Evaluation*.

### Factors Predictive of 6-month Mortality

Using univariate analysis, we identified possible risk factors for 6-month mortality (Table 4): age, Charlson Index, APACHE II score, presence of comorbidities (cardiovascular diseases, chronic renal insufficiency, COPD, type II diabetes mellitus, and solid tumors), admission to the ICU for primary causes (cardiovascular diseases and hepatobiliary/pancreatic diseases), renal replacement therapy, long-term invasive ventilation, vasopressor treatment, XDR-AB colonization, and XDR-AB infection.

**Table 4:** Univariate Analysis of Factors Associated with mortality in 6 months.

**Variables**	**Deaths at 6 months (*n* = 207)**	**Survival at 6 months (*n* = 224)**	***P*-value**	**HR (95%*CI*)**
**Age, median (IQR)**	59.92 (49,72)	55.4 (46,67.75)	<0.001	1.026 (1.017–1.035)
**Male**, ***n*** **(%)**	138 (66.7)	154 (68.8)	0.464	1.114 (0.834–1.487)
**Season**, ***n*** **(%)**				
Spring	70 (33.8)	77 (34.4)	0.879	0.978 (0.733–1.304)
Summer	45 (21.7)	58 (25.9)	0.226	0.815 (0.586–1.134)
Autumn	36 (17.4)	39 (17.4)	0.900	1.023 (0.714–1.466)
Winter	56 (27.1)	50 (222.3)	0.190	1.227 (0.903–1.668)
**Charlson Index, mean** **±** **SD**	4.630 ± 2.063	3.850 ± 2.158	<0.001	1.114 (1.056–1.175)
**Comorbidities**, ***n*** **(%)**				
Cardiovascular diseases	103 (49.8)	63 (28.1)	<0.001	1.859 (1.414–2.443)
Chronic renal insufficiency	46 (22.2)	15 (6.7)	<0.001	2.231 (1.605–3.101)
COPD	63 (30.4)	32 (14.3)	<0.001	1.897 (1.409–2.554)
Type II diabetes mellitus	56 (27.1)	45 (20.1)	0.093	1.301 (0.957–1.769)
Solid tumor	41 (19.8)	65 (29.0)	0.064	0.724 (0.514–1.019)
Hematologic malignancy	5 (2.4)	3 (1.3)	0.500	1.357 (0.559–3.296)
Current or former smoker	73 (35.3)	87 (38.8)	0.322	0.866 (0.651–1.152)
**Admission source**, ***n*** **(%)**				
Emergency department	56 (27.1)	54 (24.1)	0.503	1.111 (0.817–1.509)
Operation theater	51 (24.6)	55 (24.6)	0.981	1.004 (0.732–1.377)
Normal wards	58 (28.0)	68 (30.4)	0.719	0.946 (0.698–1.281)
Other hospital	42 (20.3)	47 (21.0)	0.737	0.944 (0.672–1.324)
**Primary reason for ICU admission**, ***n*** **(%)**				
Neurological	22 (10.6)	31 (13.8)	0.298	0.791 (0.508–1.230)
Suspected sepsis	53 (25.6)	48 (21.4)	0.363	1.156 (0.846–1.579)
Respiratory	63 (30.4)	66 (29.5)	0.871	1.025 (0.762–1.378)
Cardiovascular(excluding stroke)	40 (19.3)	25 (11.2)	0.005	1.640 (1.161–2.318)
Multiple trauma	9 (4.3)	16 (7.1)	0.217	0.656 (0.337–1.281)
Burns	2 (1.0)	5 (2.2)	0.341	0.508 (0.126–2.047)
Hepatobiliary and pancreatic	4 (1.9)	17 (7.6)	0.024	0.321 (0.119–0.864)
Gastrointestinal	7 (3.4)	12 (5.4)	0.362	0.704 (0.332–1.497)
Genitourinary	6 (2.9)	5 (2.2)	0.770	1.129 (0.501–2.542)
**XDR-AB colonization**, ***n*** **(%)**	53 (25.6)	24 (10.7)	<0.001	1.898 (1.387–2.598)
**XDR-AB infection**, ***n*** **(%)**	20 (9.7)	10 (4.5)	0.034	1.649 (1.039-2.616)
**Acuity score on admission**				
APACHE II score, mean ± SD	21.936 ± 7.406	9.125 ± 4.556	<0.001	1.107 (1.094–1.121)
**Intensity of care**, ***n*** **(%)**				
Invasive ventilation for more than 5 days	118 (57.0)	91 (40.6)	<0.001	1.641 (1.246–2.161)
Renal replacement therapy	44 (21.3)	27 (12.1)	0.005	1.616 (1.158–2.255)
Vasopressor treatment for more than 3 days	91 (44.0)	9 (4.0)	<0.001	7.849 (5.913–10.419)

*Data are number (%), median (IQR) or mean ± SD. HR, hazard ratio; COPD, chronic obstructive pulmonary disease; ICU, intensive care unit; XDR-AB, extensively drug-resistant Acinetobacter baumannii; APACHE, Acute Physiology and Chronic Health Evaluation; IQR, interquartile range; SD, standard deviation*.

Considering that XDR-AB colonization and XDR-AB infection were correlated, two multivariate Cox regression models, one for each of the variables, were created. In multivariate Cox regression model 1, we found that XDR-AB colonization was an independent risk factor for death at 6 months (HR = 1.749, 95% CI = 1.174–2.608). The other risk factors were age, high APACHE II scores, COPD, cardiovascular diseases as primary reasons for ICU admission, and extended vasopressor treatments (Table 5). In multivariate Cox regression model 2 (Supplementary Table 3), XDR-AB infection was not found to be an independent risk factor of death at 6 months.

**Table 5:** Multivariate Cox regression model for Factors Associated with mortality in 6-month (XDR-AB colonization included).

**Variables**	**HR (95%CI)**	***P*-value**
XDR-AB colonization	1.749 (1.174–2.608)	0.006
Age	1.019 (1.007–1.032)	0.002
APACHE II Score	1.127 (1.105–1.149)	<0.001
Charlson Index	0.978 (0.892–1.071)	0.629
Presence of cardiovascular diseases	1.013 (0.712–1.442)	0.942
Presence of chronic renal insufficiency	1.126 (0.755–1.677)	0.561
Presence of COPD	1.545 (1.050–2.274)	0.027
Presence of type II diabetes mellitus	0.845 (0.595–1.199)	0.345
Presence of solid tumor	0.857 (0.545–1.347)	0.503
Presence of hematologic malignancy	0.998 (0.365–2.728)	0.997
Neurological diseases as primary reason for ICU admission	0.299 (0.046–1.934)	0.205
Suspected sepsis as primary reason for ICU admission	0.258 (0.041–1.647)	0.152
Respiratory diseases as primary reason for ICU admission	0.262 (0.041–1.668)	0.156
Cardiovascular (excluding stroke) diseases as primary reason for ICU admission	3.855 (1.047–14.902)	0.045
Multiple trauma as primary reason for ICU admission	0.262 (0.037–1.865)	0.181
Burns as primary reason for ICU admission	0.217 (0.022–2.172)	0.193
Hepatobiliary and pancreatic diseases as primary reason for ICU admission	0.058 (0.007–1.045)	0.108
Gastrointestinal diseases as primary reason for ICU admission	0.179 (0.025–1.304)	0.090
Genitourinary diseases as primary reason for ICU admission	0.286 (0.037–2.191)	0.228
Invasive ventilation for more than 5 days	0.858 (0.624-1.178)	0.343
Renal replacement therapy	0.962 (0.642-1.443)	0.852
Vasopressor treatment for more than 3 days	8.295(5.710–12.051)	<0.001

*XDR-AB colonization was treated as a time-dependent variable in the Cox regression model. HR, hazard ratio; APACHE, Acute Physiology and Chronic Health Evaluation; COPD, chronic obstructive pulmonary disease; ICU, intensive care unit; XDR-AB, extensively drug-resistant Acinetobacter baumannii*.

**Figure 2:**
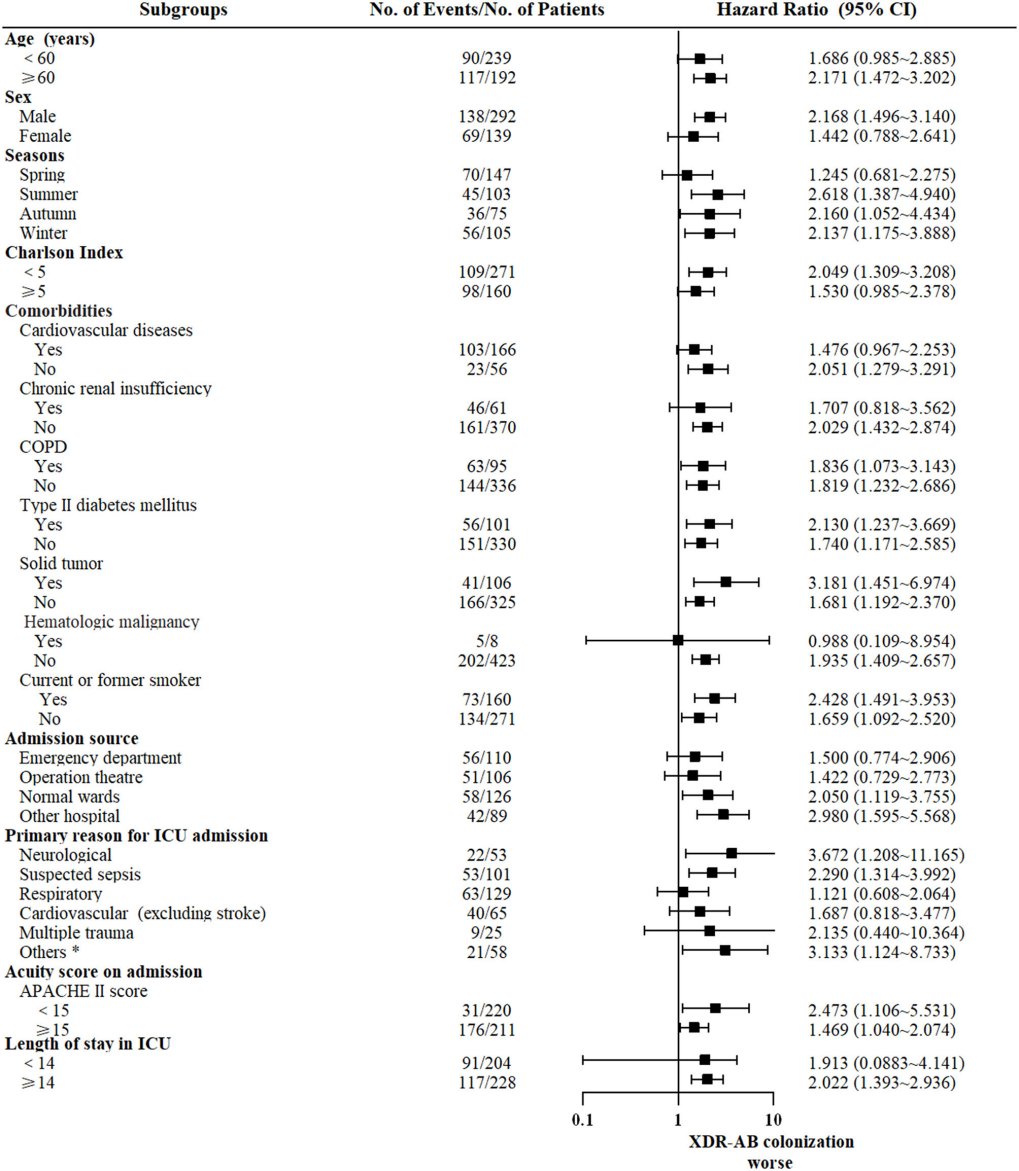
Subgroups analyses of the impact of XDR-AB colonization on mortality at 6 months. Shown are hazard ratios for 6-month survival with XDR-AB colonization compared with no colonization. *Included burns and hepatobiliary, pancreatic, gastrointestinal, and genitourinary diseases.

The impact of colonization with XDR-AB in subgroups with different baseline characteristics are presented in Figure 2. In most subgroups, the number of survival days was lower for colonized patients than for non-colonized patients.

## Discussion

*A. baumannii* is one of the most threatening nosocomial microorganisms, characterized by its capacity to survive in various environments and to develop antibiotic resistance. Drug-resistant *A. baumannii* is prevalent in several healthcare facilities in China. The rapid growth of antibiotic resistance is a considerable economic and medical burden, and is associated with higher costs, longer hospital stays, and increased in-hospital mortality (22, 23). Due to the increase in the occurrence of XDR and pan drug-resistant *A. baumannii* strains, WHO reported that there is an urgent need of novel antibiotics (24), emphasizing the importance of preventing and treating drug-resistant *A. baumannii*.

This study assessed the effect of XDR-AB colonization on the prognoses of severely ill patients. We conducted the study in two mixed adult ICUs in China, where XDR-AB is the most common pathogen in nosocomial infections. The findings revealed that XDR-AB colonization had no association with the short-term (28-day) mortality of ICU patients but contributed to a 1.75-fold increase in mortality risk at 6 months.

In our study, the overall peripheral colonization rate of XDR-AB was 18%, which is higher than that published in previous reports. An XDR-AB acquisition rate of 15.6% was observed in a Lebanese ICU (25). Further, in an American study, the colonization rate of XDR-AB among solid organ transplant patients was reported as 0.9% (26). The relatively high colonization rate in our study indicates a possible regional epidemic of XDR-AB in our ICUs.

Studies have elucidated several risk factors of XDR-AB colonization, such as previous admission to long-term healthcare facilities, invasive operations, presence of comorbidities, low socioeconomic status, and previous use of carbapenems (25, 27, 28). In this study, critical patients who were admitted with suspected sepsis and required extended invasive ventilation and longer ICU stay were more likely to be colonized with XDR-AB. There are possible reasons for this finding. First, in septic patients, immune system disorders lead to poor defensive responses, making them more susceptible to opportunistic pathogens. Second, invasive operations, such as tracheal intubation and tracheostomy, provide opportunities for pathogen colonization through wounds and invasive devices due to impairments in skin and mucosal barriers. Finally, decolonization has not been adopted in ICUs in China; therefore, long-term hospitalizations coupled with poor baseline conditions and more intensive care might increase opportunities for colonization.

There is not much information on the impact of XDR-AB colonization on patient prognosis. It has been wildly accepted that *A. baumannii* infections can lead to higher death rates. Among critically ill patients, the estimated increase in the in-hospital mortality rate due to *A. baumannii* infection ranges between 7.8 and 23%, and the attributable ICU mortality ranges from 10 to 43% (29). High mortality rates have been reported in patients infected with drug-resistant *A. baumannii* (30, 31). However, only a few studies have elucidated the association between mortality and colonization of drug-resistant strains. In one such cohort study conducted by Dautzenberg et al., it was confirmed that there is a 1.79 times higher risk of death in ICU patients colonized with carbapenemase-producing Enterobacteriaceae than in ICU patients who are not colonized (16). Currently, the association between *A. baumannii* colonization and mortality is still under debate. It has been reported that colonization with multiple drug-resistant *A. baumannii* (MDR-AB) strains upon ICU admission is related with a 1.4-fold increase in in-hospital death rate (32). Additionally, several studies have concluded that colonization and infection with *A. baumannii* is an independent risk factor of mortality (27, 33–35), without distinguishing between colonization and infection. In this study, we identified colonization using nasopharyngeal and perirectal samplings, which are generally used to identify colonization (16, 36, 37). As far as we know, this is the first research to assess the effect of XDR-AB colonization on long-term mortality in critical patients. The results revealed that XDR-AB colonization has no impact on the 28-day prognosis based on the multivariate analysis findings, but it was associated with higher mortality rates at 6 months. Thus, in our study, patients with XDR-AB colonization had a 1.75 times higher risk of death at 6 months. After adjusting for severity of illness, previous medical history, intensity of care, and other factors identified in univariate analysis, XDR-AB colonization proved to be an independent risk factor of poor long-term prognoses.

The mechanisms of bacterial colonization may hold the answer to the increased mortality observed in patients with XDR-AB colonization. In general, bacterial colonization in hosts typically occurs in several steps. First, colonizers enter the nasopharynx and escape from the mucus, and following this, they attach themselves and adhere to epithelial cells. At the colonization loci, pathogens acquire the nutrition required to grow and to proliferate via various pathways of carbohydrate transportation and utilization (38, 39), and eventually evade host immune responses and achieve persistence. In the case of *A. baumannii*, colonization starts with pili-mediated twitching, and adherence is assisted by cell surface hydrophobicity and biofilm-associated proteins (38, 40). Insufficient nutrient availability in the upper respiratory tract promotes the transformation from motion to colonization (41). Additionally, persistent invasion of the host immune system is facilitated by capsules, immunoglobulin-targeted proteases, and the biofilm (42–44). The colonization is also dependent on the establishment of balance between hosts and pathogens: Immunocompetent hosts tend to coexist with the microorganisms, while infection occurs in deteriorated immune systems.

The association between *A. baumannii* colonization and mortality could be explained by several mechanisms. For example, virulence factors for *A. baumannii*, particularly porins, can cause cytotoxicity and induce immune responses. Porins, including OmpA, Omp34, and carO, exist on the outer membranes of *A. baumannii*. They have been found to be correlated with antimicrobial resistance, inflammatory responses, and cell death (45, 46). OmpA is also found in secreted membrane vesicles, and increase in the density of OmpA has been proven to be associated with high mortality (47). Additionally, spread of colonized pathogens into the lower respiratory tract, or other sterile locations, can lead to increased risk of diffuse infections (48). Moreover, interactions between colonizers can result in coinfections or induction of antibiotic resistance even without antibiotic exposure. In fact, this appears to be a prominent mechanism in bacteria with extensive drug resistance.

Increase in mortality caused by XDR-AB colonization indicates the need for essential surveillance during the early stage and efficient measures of decolonization.

A meta-analysis reported that decolonization reduces infection caused by multidrug-resistant gram-negative bacteria when combined with standard care, especially in Europe, where decolonization has been widely applied as an infection prevention and control strategy (49). However, considering that there is a lack of effective antibiotics, it is challenging to eliminate XDR-AB. It has been reported that daily whole-body bathing with chlorhexidine was efficient in eliminating MDR-AB from the skin (50) and was helpful in reducing bloodstream infections in patients colonized with XDR gram-negative bacilli (51). Thus, this might be a feasible approach to decolonize XDR-AB. Decontamination of the alimentary tract with polymyxin E and tobramycin was effective in patients colonized with MDR-AB (37, 52–54). However, there is no clinical evidence about systematic antibiotics for the decolonization of XDR-AB. Additionally, standard nosocomial care is of vital importance, e.g., hand hygiene, exposure precautions, conventional screening, and environmental sterilization, especially in wards with highly prevalent strains (3). However, according to the guidelines established by the European Committee on Infection Control (EUCIC), there is not enough information about the decolonization of carbapenem-resistant *Acinetobacter baumannii* (CRAB) (55). Our data provide some evidence supporting the need for decolonization, but further interventional studies are required for strategy development and efficacy validation.

It has been acknowledged that in critical patients, colonization with gram-negative bacteria contributes to more nosocomial infections (56). The risk of developing subsequent *A. baumannii* infections is 8.4 times higher in patients colonized with CRAB (36). In this study, the incidence of XDR-AB infection is higher in colonized patients than in non-colonized ones. We also observed higher use of colistin or tigecycline after detection of XDR-AB colonization. Multivariate Cox regression analysis showed that XDR-AB infection is not an independent risk factor for death at 6 months. This implies that the increase in the 6-month mortality of colonized patients was not caused by subsequent XDR-AB infection during admission.

Subgroup analyses of 6-month mortality rates were performed in patients with different characteristics, and the results were consistent. Patients colonized with XDR-AB had the worse prognosis (with HR >1) in all the groups; however, some of the subgroups did not show statistically significant differences. Colonization with XDR-AB was recognized as a risk factor of 6-month mortality regardless of age, admission season, comorbidity, and APACHE II scores upon admission. In addition, XDR-AB colonization was worse in ICU patients who were hospitalized for more than 14 days (HR = 2.022, 95% CI = 1.393–2.936). This indicates the harmful effect of XDR-AB colonization on critical patients with prolonged ICU stay.

Our study had some limitations. First, there is no information on the effect of colonization with drug-sensitive *A. baumannii* or MDR-AB on patient prognosis. Approximately 95% of the *A. baumannii* strains isolated were XDR strains, and sensitive and multidrug-resistant strains were rare. Second, we assumed in colonized patients, the XDR-AB colonization persisted at discharge, because decolonization measures were not performed. Third, information about XDR-AB infection from the patients' discharge to 6 months after discharge could not be obtained; therefore, its influence on 6-month mortality may have been underestimated. Fourth, we did not determine cause-specific survival as we were unable to obtain follow-up data for all the patients. Patients were re-admitted to other hospitals that used different information systems, so it was difficult to obtain their medical records after discharge.

## Conclusions

Our study provided some evidence for the impact of XDR-AB colonization on the prognosis of critically ill patients. XDR-AB colonization had no effect on the short-term mortality of ICU patients; however, it increased the 6-month mortality rates by 1.75-fold. In the future, efficient prevention and control methods must be investigated for the management of nosocomial drug-resistant *A. baumannii*.

## Data Availability Statement

The raw data supporting the conclusions of this article will be made available by the authors, without undue reservation.

## Ethics Statement

The studies involving human participants were reviewed and approved by the Ethics Committee of Qilu Hospital, Shandong University. Written informed consent for participation was not required for this study in accordance with the national legislation and the institutional requirements.

## Author Contributions

YZ contributed to data interpretation and manuscript preparing. NX contributed to information collection and data analysis. JP contributed to manuscript preparing. HH and HY helped data interpretation. WQ, HZ, and WL helped data collection. HW and YC contributed to study design and manuscript writing. All authors approved the final manuscript and are responsible for the content.

## Conflict of Interest

The authors declare that the research was conducted in the absence of any commercial or financial relationships that could be construed as a potential conflict of interest.

## Abbreviations

APACHE: Acute Physiology and Chronic Health Evaluation
CCI: Charlson Comorbidity Index
CI: confidence interval
CLSI: American Clinical and Laboratory Standards Institute
COPD: chronic obstructive pulmonary disease
CRAB: carbapenem-resistant *Acinetobacter baumannii*
EUCIC: European Committee on Infection Control
HR: hazard ratio
ICU: intensive care unit
IQR: interquartile range
MDR-AB: multiple drug-resistant *Acinetobacter baumannii*
OR: odds ratio
SD: standard deviation
WHO: World Health Organization
XDR-AB: extensively drug-resistant *Acinetobacter baumannii*.
