# Supplementary material for: Colonization With Extensively Drug-Resistant Acinetobacter baumannii and Prognosis in Critically Ill Patients: An Observational Cohort Study
Source: Front Med (Lausanne). 2021 Apr 30;8:667776. doi: 10.3389/fmed.2021.667776 (PMC8119758; doi:10.3389/fmed.2021.667776)
Supplement: Supplementary file 1 [file Table_1.DOCX]

Supplementary Materials

**Table S1. Proportional Hazards Assumption for Potential Time-dependent Variables**

| **Variables** | **B** | **SE** | **Wald** | ***P* value** |
| --- | --- | --- | --- | --- |
| XDR-AB colonization | 0.458 | 0.175 | 6.883 | 0.009 |
| Age | 0.020 | 0.005 | 17.581 | 0.056 |
| Presence of cardiovascular diseases | 0.310 | 0.146 | 4.501 | 0.134 |
| Presence of chronic renal insufficiency | 0.306 | 0.180 | 2.890 | 0.089 |
| Presence of COPD | 0.192 | 0.160 | 1.449 | 0.229 |
| Presence of type II diabetes mellitus | 0.136 | 0.165 | 0.675 | 0.411 |
| Presence of solid tumor | -0.324 | 0.178 | 3.288 | 0.070 |
| Presence of hematologic malignancy | 0.483 | 0.530 | 0.832 | 0.362 |
| Current or former smoker | 0.192 | 0.155 | 1.532 | 0.216 |
| Respiratory diseases | -0.008 | 0.157 | 0.003 | 0.958 |
| Suspected sepsis | 0.128 | 0.168 | 0.576 | 0.448 |
| Neurological diseases | 0.007 | 0.237 | 0.001 | 0.975 |
| Multiple trauma | 0.121 | 0.368 | 0.109 | 0.742 |
| Cardiovascular (excluding stroke) diseases | -0.043 | 0.183 | 0.056 | 0.813 |
| Burns | 0.117 | 0.770 | 0.023 | 0.879 |
| Hepatobiliary and pancreatic diseases | -0.513 | 0.504 | 1.034 | 0.309 |
| Gastrointestinal diseases | -0.163 | 0.396 | 0.169 | 0.681 |
| Genitourinary diseases | 0.187 | 0.447 | 0.174 | 0.676 |
| Charlson Index | -0.003 | 0.028 | 0.011 | 0.917 |
| Spring | -0.068 | 0.153 | 0.201 | 0.654 |
| Summer | 0.261 | 0.185 | 1.976 | 0.160 |
| Autumn | -0.032 | 0.191 | 0.029 | 0.865 |
| Winter | -0.102 | 0.162 | 0.397 | 0.529 |
| Renal replacement therapy | -0.065 | 0.175 | 0.139 | 0.710 |
| Invasive ventilation for more than five days | -0.070 | 0.148 | 0.223 | 0.636 |
| Vasopressor treatment for more than three days | -1.459 | 0.286 | 25.997 | 0.100 |
| XDR-AB infection | -0.331 | 1.026 | 0.104 | 0.747 |

XDR-AB, extensively drug-resistant *Acinetobacter baumannii*; COPD, chronic obstructive pulmonary disease.

**Table S2. Multivariate Analysis of Factors Associated with XDR-AB Colonization**

| **Variables** | **OR (95% *CI*)** | ***P* value** |
| --- | --- | --- |
| Presence of cardiovascular diseases | 1.580 (0.826-3.023) | 0.167 |
| Presence of COPD | 1.434 (0.697-2.953) | 0.327 |
| Presence of type II diabetes mellitus | 1.501 (0.777-2.897) | 0.226 |
| Presence of solid tumor | 0.602 (0.267-1.355) | 0.220 |
| Current or former smoker | 1.328 (0.728-2.422) | 0.355 |
| Admission from other hospital | 1.698 (0.872-3.307) | 0.120 |
| Neurological diseases as primary reason for ICU admission | 0.901 (0.309-2.622) | 0.848 |
| Suspected sepsis as primary reason for ICU admission | 2.287 (1.185-4.413) | 0.014 |
| Burn as primary reason for ICU admission | 5.792 (0.954-35.166) | 0.056 |
| Hepatobiliary and pancreatic diseases as primary reason for ICU admission | 0.336 (0.037-3.049) | 0.332 |
| APACHE II score | 1.019 (0.983-1.056) | 0.316 |
| Renal replacement therapy | 1.417 (0.678-2.965) | 0.354 |
| Invasive ventilation for more than 5 days | 4.753 (2.370-9.528) | ＜0.001 |
| Vasopressor treatment for more than 3 days | 1.916 (0.965-3.805) | 0.063 |
| Length of stay in ICU | 1.043 (1.023-1.063) | ＜0.001 |

XDR-AB, extensively drug-resistant *Acinetobacter baumannii*; OR, odds ratio; CI, confidence interval; COPD, chronic obstructive pulmonary disease; ICU, intensive care unit; APACHE, Acute Physiology and Chronic Health Evaluation.

**Table S3. Multivariate Cox regression model for Factors Associated with mortality in 6-month (XDR-AB infection included)**

| **Variables** | **HR (95% *CI*)** | ***P* value** |
| --- | --- | --- |
| Age | 1.019 (1.007-1.031) | 0.002 |
| APACHE II Score | 1.130 (1.107-1.153) | ＜0.001 |
| Charlson Index | 0.981 (0.894-1.007) | 0.685 |
| Presence of cardiovascular diseases | 1.034 (0.725-1.476) | 0.852 |
| Presence of chronic renal insufficiency | 1.213 (0.814-1.807) | 0.343 |
| Presence of COPD | 1.400 (0.957-2.049) | 0.083 |
| Presence of type II diabetes mellitus | 0.812 (0.576-1.145) | 0.235 |
| Presence of solid tumor | 0.860 (0.547-1.351) | 0.513 |
| Presence of hematologic malignancy | 1.060 (0.391-2.877) | 0.909 |
| Neurological diseases as primary reason for ICU admission | 0.269 (0.041-1.785) | 0.174 |
| Suspected sepsis as primary reason for ICU admission | 0.219 (0.034-1.426) | 0.112 |
| Respiratory diseases as primary reason for ICU admission | 0.251 (0.038-1.634) | 0.148 |
| Cardiovascular (excluding stroke) diseases as primary reason for ICU admission | 6.990 (1.042-26.911) | 0.045 |
| Multiple trauma as primary reason for ICU admission | 0.246 (0.034-1.786) | 0.166 |
| Burns as primary reason for ICU admission | 0.264 (0.025-2.781) | 0.267 |
| Hepatobiliary and pancreatic diseases as primary reason for ICU admission | 0.170 (0.863-2.221) | 0.170 |
| Gastrointestinal diseases as primary reason for ICU admission | 0.175 (0.024-1.301) | 0.089 |
| Genitourinary diseases as primary reason for ICU admission | 0.322 (0.041-2.542) | 0.282 |
| Invasive ventilation for more than five days | 0.790 (0.580-1.075) | 0.133 |
| Renal replacement therapy | 0.893 (0.600-1.330) | 0.578 |
| Vasopressor treatment for more than three days | 14.838 (10.042-21.923) | ＜0.001 |
| XDR-AB infection | 0.698 (0.410-1.188) | 0.185 |

HR, hazard ratio; CI, confidence interval; APACHE, Acute Physiology and Chronic Health Evaluation; COPD, chronic obstructive pulmonary disease; ICU, intensive care unit; XDR-AB, extensively drug-resistant *Acinetobacter baumannii*.
